# Postpandemic masking practices among health care personnel: beliefs, barriers, and opportunities for improving adherence in clinical settings

**DOI:** 10.1017/ash.2025.10265

**Published:** 2026-01-14

**Authors:** Karina Ohri, Samantha E. Hanley, Nicholas Allis, Telisa Stewart, Mitchell Brodey, Paul Suits, Stephen J. Thomas, Jana Shaw

**Affiliations:** 1 Norton College of Medicine, SUNY Upstate Medical University, Syracuse, NY, USA; 2 Department of Public Health and Preventive Medicine, SUNY Upstate Medical University, Syracuse, NY, USA; 3 Department of Internal Medicine, SUNY Upstate Community Hospital, Syracuse, NY, USA; 4 Infection Prevention, SUNY Upstate Medical University, Syracuse, NY, USA; 5 Upstate Global Health Institute, Syracuse, NY, USA; 6 Upstate Golisano Children’s Hospital Epidemiologist, Division of Infectious Diseases, Department of Pediatrics, SUNY Upstate Medical University, Syracuse, NY, USA

## Abstract

**Objective:**

This study examines gaps in mask-related behaviors, beliefs, and perceptions among healthcare personnel (HCP), investigates the influence of vaccination status on masking practices, and identifies opportunities to enhance adherence in clinical settings.

**Methods:**

A survey was conducted among HCP providing direct patient care at State University of New York Upstate Medical University from November 2024 to January 2025.

**Results:**

A total of 655 HCP responded to the survey. Of these, 335 (51.1%) reported being up to date on coronavirus (COVID-19) vaccination, and 501 (76.5%) intended to receive the 2024 – 2025 influenza vaccine. Majority believed masking protects them (*n* = 381, 61%) and others (*n* = 400, 64.6%), perceived the workplace as carrying a high respiratory risk (*n* = 366, 58.6%), and believed patient masking offers protective benefits (*n* = 359, 57.4%). Self-reported masking rates were highest when participants were sick or symptomatic (*n* = 477, 72.8%) and lowest during respiratory specimen collection (*n* = 233, 35.6%). High adherence was observed when participants were symptomatic, among those who were up to date on their COVID-19 vaccination (*n* = 276, 82.4%) and who intended to receive the 2024 – 2025 influenza vaccine (*n* = 407, 81.2%).

**Conclusion:**

These findings suggest self-reported masking adherence remains suboptimal, reflecting low perceived risk of COVID-19 infection in the postpandemic period. Targeted interventions highlighting masking as an important component of broader measures including vaccination, hand hygiene, and ventilation are needed to enhance hospital infection prevention and control.

## Introduction

Hospital-acquired respiratory infections (HARI) represent a significant health concern. Estimates of hospital-acquired coronavirus (COVID-19) infections among patients in the United States vary widely, ranging from 0.02% to 65.0%.^1^ In addition, compared to community acquired respiratory syncytial virus, nearly half of hospital-acquired cases required a higher level of care at discharge.^2^ Healthcare personnel (HCP) also face an increased HARI risk. A systematic review found that HCP have a higher risk of acquiring influenza infection than the general population, partly due to asymptomatic cases.^3^ A 2021 study found 90% of HCP infected with influenza were asymptomatic or mildly symptomatic, posing a potential transmission risk.^4^ During the COVID-19 pandemic, HCP experienced a 12-fold higher infection risk than the general community.^5^

Face masks reduce transmission of respiratory pathogens.^6–9^ At Singapore’s largest healthcare system, universal masking helped reduce respiratory viral infection from 9.69 to 0.83 cases per 10,000 patient days.^10^ In 2022, the end of the mask mandate at the Children’s Health System of Texas coincided with an increase in respiratory infections.^11^ Among HCP, in 2020, universal masking reduced SARS-COV-2 positivity from 14.65% to 11.46%.^12^ Furthermore, in 2022, Michigan’s COVID-19 epicenter implemented a universal masking policy that provided surgical masks to team members working in hospital facilities. Implementation of this policy resulted in a reduction of COVID-19 transmission, by half, every 10.5 to 13.5 days.^13^ Despite this, masking adherence remains low.

In early 2020, at the start of the COVID-19 pandemic, universal masking mandates were implemented in hospitals to curb SARS-COV-2. Since then, the Centers for Disease Control and Prevention (CDC) recommend healthcare facilities use a “risk-based assessment” when masking to prevent transmission of COVID-19. However, the CDC recommends universal masking under specific circumstances such as outbreaks or entering rooms with suspected or confirmed COVID-19 cases.^14^ With the end of the public health emergency, healthcare centers have withdrawn universal masking policy, recommending it only in limited circumstances, such as working with patients with contagious respiratory infections.^15^ HCP remain at an elevated risk for HARI, making it essential to understand their mask-wearing attitudes and practices to promote consistent use.

We conducted a cross-sectional online survey of HCP in a large tertiary care academic setting with direct patient contact to assess mask-wearing behaviors, beliefs, and perceptions. We also examined the influence of influenza and COVID-19 vaccination status on mask wearing behaviors. This study aims to inform quality improvement initiatives that enhance mask usage, reduce transmissions of HARIs, and improve patient outcomes.

## Methods

### Study population

The State University of New York (SUNY) Upstate Medical University in Syracuse, NY, the region’s only academic center, serves 1.8 million people across 17 counties. HCP with direct patient care duties were included in the survey. The SUNY Upstate Institutional Review Board determined this project to be exempt from IRB review (#2237479–20).

### Survey administration

An anonymous online REDCap survey was conducted from November 2024 to January 2025 and distributing through institutional weekly emails. Participation was voluntary, with an optional $100 raffle.

### Survey instrument development

The survey was developed by a multidisciplinary team with expertise in infectious diseases, epidemiology, and public health and through a systematic process incorporating literature review, expert consultation, and pilot testing.

The survey was organized into four main sections:

1. *Demographics*: Age, gender, race, ethnicity, role in the hospital, and work setting.
2. *Attitudes and perceptions*: Beliefs about mask efficacy, comfort, and institutional policies in general.
3. *Behaviors:* Self-reported adherence when collecting respiratory specimen, entering patient rooms under droplet precautions, caring for patients with respiratory symptoms, and when respondents were themselves symptomatic. Respiratory specimen collection included testing of symptomatic and asymptomatic patients as part of the hospital’s universal COVID-19 admission testing policy.
4. *Vaccination information*: Self-reported COVID-19 and influenza vaccination status and its influence on mask-wearing behavior.

The survey consisted of 23 questions, primarily comprising closed-ended questions using Likert scales, multiple-choice, and yes/no formats. An “Other” option, and one open-ended question were included to elicit additional feedback, which was thematically analyzed. The survey was pilot tested with a small group of clinical staff to ensure clarity and face validity before distribution.

### Data analysis

Records initially labeled as “incomplete” by REDCap were manually reviewed. Entries that included only test records or responses limited to the first or demographic questions, were removed. Data cleaning included reassigning “Other” responses to predefined categories if the text exactly matched existing options.

Descriptive statistics summarized participant characteristics, attitudes, and behaviors. Stratified analyses examined differences by role and vaccination status for COVID-19 and intent to vaccinate against influenza for the 2024 – 2025 season (October 2024-May 2025). Likert responses were collapsed into three categories: Agree, Unsure, and Disagree. “Other” role responses were reviewed and re-coded into a new set of role codes that included original selections and written “Other” responses. All roles were recoded using this updated scheme. “High adherence” was defined as ≥ 75% compliance and “low adherence” as < 75%.^16^ Group differences were analyzed using χ^2^ tests and Pearson in SPSS (V25). Qualitative data were collected from three questions that included an “Other” option, as well as from an open-ended question soliciting participant feedback. An Inductive Coding approach was used in ATLAS.ti.

## Results

### Participant characteristics

There was a total of 746 participants, of which 655 met the inclusion criteria and were included in the study. Most participants were white (*n* = 567, 86.6%) and female (*n* = 527, 80.5%), with a mean age of 44 years (SD = 12). Regarding professional role, 237 (36.2%) were registered nurses, 137 (20.9%) scientists/physicians (including research staff, medical students, and physicians), 122 (18.6%) allied health professionals (AHPs) (physical therapy, occupational therapy, psychiatry, respiratory services, and clinical support staff), 73 (11.1%) Master’s-level clinicians (nurse practitioners, physician’s assistants, and social workers/case management), 62 (9.5%) ancillary services (ultrasound, radiologic technicians, clerical, dietary, phlebotomy, unit support, registration, clinical support, and environmental services), and 24 (3.7%) other hospital roles. Overall, 335 (51.1%) respondents reported to be up to date on COVID-19 vaccination, and 501 (76.5%) intended to receive the 2024 – 2025 influenza vaccine. Table 1 contains further demographic characteristics and vaccination details.

**Table 1.** Participant’s demographic characteristics and vaccination status

Role	*n* (%)	Female *n* (%)	Male *n* (%)	Mean age *n* (SD)	White *n* (%)	Black or African American *n* (%)	Asian *n* (%)	Other^f^ *n* (%)	Are you up to date on your COVID-19 Vaccination?^g^		For this coming winter (2024 – 2025), do you intend to be vaccinated against seasonal influenza?	
									Yes *n* (%)	No/Unsure *n* (%)	Yes *n* (%)	No/Unsure *n* (%)
Total respondents	655	527 (80.5)	100 (15.3)	44.08 (12.0)	567 (86.6)	19 (2.9)	20 (3.1)	49 (7.5)	335 (51.1)	265 (40.5)	501 (76.5)	99 (15.1)
Registered nurses	237 (36.2)	211 (89.0)	17 (36.2)	43.35 (11.2)	218 (92.0)	5 (2.1)	2 (.8)	12 (5.1)	102 (43.0)	116 (48.9)	181 (76.4)	37 (15.9)
Scientists/ physicians^a^	137 (20.9)	78 (56.9)	50 (20.9)	44.50 (14.1)	105 (76.6)	3 (2.2)	15 (10.9)	14 (10.2)	96 (70.1)	33 (24.1)	123 (89.8)	6 (4.4)
Allied health professionals^b^	122 (18.6)	99 (81.1)	18 (18.6)	45.79 (11.0)	105 (86.1)	5 (4.1)	1 (3.7)	11 (9.0)	63 (51.6)	46 (37.7)	81 (66.4)	27 (26.1)
Master’s-level clinicians^c^	73 (11.1)	69 (58.7)	4 (5.5)	45.62 (11.8)	67 (91.8)	1 (1.4)	1 (1.4)	4 (5.5)	37 (50.7)	30 (41.1)	63 (86.3)	4 (5.5)
Ancillary services^d^	62 (9.5)	49 (79.0)	9 (14.5)	42.24 (11.8)	53.7 (80.6)	5 (8.1)	1 (1.6)	6 (9.7)	26 (41.9)	29 (46.8)	33 (53.2)	23 (32.3)
Other^e^	24 (3.7)	21 (19.3)	3.7 (8.3)	40.25 (11.0)	22 (86.6)	0 (0)	0 (0)	2 (8.3)	11 (45.8)	11 (45.8)	20 (83.8)	2 (8.4)
*P* value^h^									.004		< .001	

a Scientists/physicians: Includes research staff, medical students, and physician.

b Allied health professionals: Includes physical therapy, occupational therapy, psychiatry, and respiratory services, clinical support staff (CNA/LPN/HCT/MA).

c Master’s-level clinicians: Includes nurse practitioners, physician’s assistants, and social workers/case management.

d Ancillary services: Includes ultrasound, ct tech, eeg tech, radiologic tech, clerical, dietary, phlebotomy, unit support, registration, clinical support, and environmental services.

e Other: Public safety, spiritual care, laboratory, informational technology, and pharmacy services, Phlebotomy, or other.

f American Indian, Alaska Native, or other.

g Self-reported vaccination status.

h Derived from Pearson χ^2^.

### Attitudes and perceptions to masking

**Figure 1.**
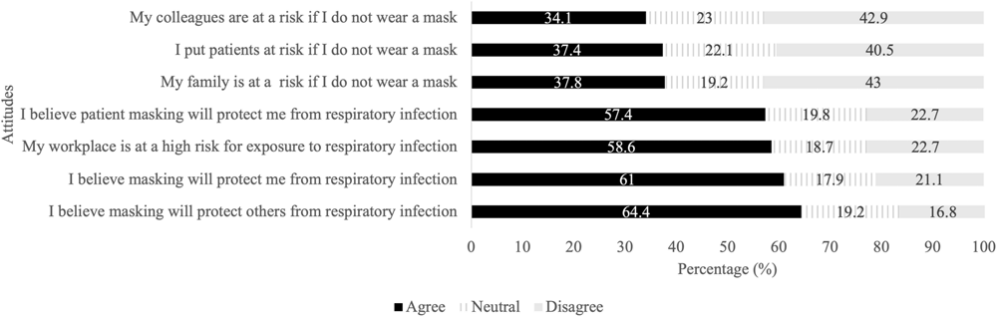
Participants’ beliefs regarding masking effectiveness and workplace infection risk (*n* = 625). The graph shows the percentage of participants who selected “Agree” “Disagree” or “Neutral” for each statement assessing attitudes.

Participants were asked a series of questions regarding their beliefs about masking in general. The most frequently endorsed statements were: “Masking protects others from respiratory infection” (*n* = 400, 64.4%), “Masking protects me from respiratory infection” (*n* = 381, 61.0%), “My workplace is at high risk for exposure to respiratory infection” (*n* = 366, 58.6%), and “Patient masking will protect me from respiratory infection” (*n* = 359, 57.4%). Statements most commonly disagreed with included: “My family is at a risk if I do not wear a mask” (*n* = 269, 43.0%), “My colleagues are at a risk if I do not wear a mask” (*n* = 268, 42.9%), and “I put patients at a risk if they do not wear a mask” (*n* = 253, 40.5%) (Figure 1).

Participants were also asked about their views on mask policy. When asked whether ear loop mask policy played a role in protecting people during the COVID-19 pandemic, 442 (67.5%) participants agreed. When asked whether visitors should wear masks while visiting the hospital during respiratory season (defined as the period from fall to spring), 347 (53.0%) participants agreed. Additional stratification by role for each of these questions is shown in Table 2. When asked whether ear loop masking should be part of routine clinical encounters during respiratory season, agreement (*n* = 276, 42.1%), varied by role. Scientists and physicians reported the highest support for routine masking (*n* = 77, 56.2%), followed by Master’s level clinicians (*n* = 32, 43.8%), ancillary services staff (*n* = 25, 40.3%), whereas registered nurses (*n* = 89, 37.6%) and AHPs (*n* = 42, 34.4%) reported the lowest (Table 2). In discussing the preferred ear loop mask policy for health care staff providing direct patient care, respondents were more likely to choose “Mandated based on patient isolation status” (*n* = 293, 44.7%) and least likely to choose “During peak season (ie, fall and winter months)” (*n* = 163, 24.9%) (Table 3).

**Table 2.** Relationship between participant role and beliefs regarding masking

Role	Do you believe wearing ear loop mask played a role in protecting people during the COVID-19 pandemic?			Do you believe ear loop masking should be a part of routine patient encounters during respiratory season?			Do you believe visitors should wear masks while visiting patients in the hospital during respiratory season?		
	Yes *n* (%)	No *n* (%)	Unsure *n* (%)	Yes *n* (%)	No *n* (%)	Unsure *n* (%)	Yes *n* (%)	No *n* (%)	Unsure *n* (%)
Registered nurses	146 (61.6)	67 (28.3)	24 (10.1)	89 (37.6)	126 (53.2)	22 (9.3)	117 (49.4)	97 (40.9)	23 (9.7)
Scientists/physicians^a^	119 (86.9)	12 (8.8)	6 (4.4)	77 (56.2)	37 (27.0)	23 (16.8)	85 (62.0)	37 (27.0)	15 (10.9)
Allied health professionals^b^	76 (62.3)	37 (30.3)	9 (7.4)	42 (34.4)	67 (54.9)	13 (10.7)	60 (49.2)	46 (37.7)	16 (13.1)
Master’s-level clinicians^c^	57 (78.1)	9 (12.3)	7 (9.6)	32 (43.8)	30 (41.1)	11 (15.1)	39 (53.4)	23 (31.5)	11 (15.1)
Ancillary services^d^	30 (48.4)	21 (33.9)	11 (17.7)	25 (40.3)	30 (48.4)	7 (11.3)	30 (48.4)	26 (41.9)	6 (9.7)
Other^e^	14 (58.3)	7 (29.2)	3 (12.5)	11 (45.8)	10 (41.7)	3 (12.5)	16 (66.7)	8 (33.3)	0 (0)
*P* value	< .001			< .001			.114		

a Scientists/physicians: Includes research staff, medical students, and physician.

b Allied health professionals: Includes physical therapy, occupational therapy, psychiatry, and respiratory services, clinical support staff (CNA/LPN/HCT/MA).

c Master’s-level clinicians: Includes nurse practitioners, physician’s assistants, and social workers/case management.

d Ancillary services: Includes ultrasound, ct tech, eeg tech, radiologic tech, clerical, dietary, phlebotomy, unit support, registration, clinical support, and environmental services.

e Other: Public safety, spiritual care, laboratory, informational technology, and pharmacy services, phlebotomy, or other.

**Table 3.** Participant preferred ear loop mask policy

Policy	Yes *n* (%)	No *n* (%)
Personal choice	271 (41.4)	384 (58.6)
Mandated based on patient’s isolation status	293 (44.7)	362 (55.3)
Mandated dependent on level of community transmission of viral pathogens (i.e., Influenza, COVID-19, RSV)	228 (34.8)	427 (65.2)
Mandated, dependent on vaccination status	196 (29.9)	459 (70.1)
During peak season (i.e. fall and winter months)	163 (24.9)	492 (75.1)
I am not sure	10 (1.5)	645 (98.5)

### Behaviors and barriers

To assess mask-wearing behaviors, participants were asked to indicate in which situations they would choose to mask. Self-reported masking rates were highest when participants were sick or showing respiratory symptoms (*n* = 477, 72.8%) and when entering patient rooms under droplet precautions (*n* = 449, 68.5%), moderate when working with patients with respiratory symptoms (*n* = 404, 61.8%), and lowest when collecting respiratory specimens (*n* = 233, 35.6%). Masking upon entering patient rooms under droplet precaution was most common among registered nurses (*n* = 186, 78.5%), the only group achieving high adherence (≥ 75%), followed by scientists/physicians (*n* = 99, 72.3%), Master’s-level clinicians (*n* = 46, 63.0%), AHPs (*n* = 76, 62.3%), and ancillary services (*n* = 26, 41.9%) (Table 4). During respiratory sample collection, 50.2% of registered nurses (*n* = 119) and 54.7% (*n* = 75) of scientists/physicians reported wearing masks. In contrast, the majority of AHPs (*n* = 99, 81.1%), Master’s-level clinicians (*n* = 54, 74.0%), and ancillary services staff (*n* = 56, 90.3%) reported not wearing masks during respiratory sample collection (Table 4).

**Table 4.** Relationship between participant role and masking behaviors across clinical scenarios

	In which of the following situations do you wear a mask?									
	When collecting a respiratory specimen^g^		When entering patient rooms under droplet precautions		When working with a patient with respiratory symptoms		When I am sick or showing respiratory symptoms		I never wear a mask	
Role	Yes *n* (%)	No *n* (%)	Yes *n* (%)	No *n* (%)	Yes *n* (%)	No *n* (%)	Yes *n* (%)	No *n* (%)	Yes *n* (%)	No *n* (%)
Registered nurses	118 (49.8)	119 (50.2)	186 (78.5)	51 (21.5)	151 (63.7)	86 (36.3)	180 (75.9)	57 (24.1)	8 (3.4)	229 (96.6)
Scientists/ physicians^a^	62 (45.3)	75 (54.7)	99 (72.3)	38 (27.7)	93 (67.9)	44 (32.1)	105 (76.6)	32 (23.4)	6 (4.4)	131 (95.6)
Allied health professionals^b^	23 (18.9)	99 (81.1)	76 (62.3)	46 (37.7)	73 (59.8)	49 (40.2)	83 (68.0)	39 (32.0)	7 (5.7)	115 (94.3)
Master’s-level clinicians^c^	19 (26.0)	54 (74.0)	46 (63.0)	27 (37.0)	44 (60.3)	29 (39.7)	55 (75.3)	18 (24.7)	2 (2.7)	71 (97.3)
Ancillary services^d^	6 (9.7)	56 (90.3)	26 (41.9)	36 (58.1)	29 (46.8)	33 (53.2)	37 (59.7)	25 (40.3)	7 (11.3)	55 (88.7)
Other^e^	5 (20.8)	19 (79.2)	16 (66.7)	8 (33.3)	14 (58.3)	10 (41.7)	17 (70.8)	7 (29.2)	1 (4.2)	23 (95.8)
*P* value^f^	< .001		< .001		.12		.10		.17	

a Scientists/physicians: Includes research staff, medical students, and physicians.

b Allied health professionals: Includes physical therapy, occupational therapy, psychiatry, and respiratory services, clinical support staff (CNA/LPN/HCT/MA)

c Master’s-level clinicians: Includes nurse practitioners, physician’s assistants, and social workers/case management.

d Ancillary services: Includes ultrasound, ct tech, eeg tech, radiologic tech, clerical, dietary, phlebotomy, unit support, registration, clinical support, and environmental services.

e Other: Public safety, spiritual care, laboratory, informational technology, and pharmacy services, Phlebotomy, or other.

f Derived from Pearson χ^2^.

g Included testing for all patients, regardless of symptoms.

The leaders most frequently trusted included the CDC (*n* = 489, 74.7%), the National Institute of Health (*n* = 353, 53.9%), and hospital leadership (*n* = 289, 41.1%). When asked who influences their masking decision, the most frequent response was “No one” (*n* = 399, 60.9%). Among 106 responses under “other,” top emergent themes included: health professionals and leaders (*n* = 27, 25.5%), scientific information, national health guidance (*n* = 19, 17.9%), and themselves (*n* = 18, 16.9%).

Participants identified numerous barriers to masking with the top three including skin irritation (*n* = 206, 31.4%), difficulty breathing (*n* = 196, 30%), and vision interference (*n* = 190, 29%). Under “Other,” the top emergent themes included no issues wearing a mask (*n* = 40, 25.8%), concerns with communication (*n* = 38, 24.5%), and interference with human connection and relationship building (*n* = 31, 20%).

### Influence of vaccination status

A comparative analysis was conducted based on participants’ vaccination status and associated masking behaviors under different clinical scenarios. Overall, COVID-19 and influenza vaccination status had limited practical impact on masking behavior across clinical scenarios (Table 5). Masking varied by context, with the lowest rates reported among participants not intending to receive the influenza vaccine when collecting respiratory specimens (*n* = 23, 32.4%) and the highest among those up to date on COVID-19 vaccination when they were ill or experiencing respiratory symptoms (*n* = 276, 82.4%) (Table 5).

**Table 5.** Association between vaccination status and masking behavior in clinical scenarios

Masking behavior		Are you up to date on COVID-19 vaccination^a^?				For this coming winter (2024 – 2025), do you intend to be vaccinated against seasonal influenza?			
In which of the following situations do you wear a mask?		Yes *n* (%)	No *n* (%)	Unsure *n* (%)	*P* value^b^	Yes *n* (%)	No *n* (%)	Unsure *n* (%)	*P* value^b^
When collecting a respiratory specimen^c^	Yes	136 (40.6)	85 (37.4)	10 (26.3)	< .001	196 (39.1)	23 (32.4)	12 (42.9)	< .001
	No	199 (59.4)	142 (62.6)	28 (73.7)		305 (60.9)	48 (67.6)	16 (57.1)	
When entering patient rooms under droplet precautions	Yes	249 (74.3)	170 (74.9)	22 (57.9)	< .001	375 (74.9)	50 (70.4)	16 (57.1)	< .001
	No	86 (25.7)	57 (25.1)	16 (42.1)		126 (25.1)	21 (29.6)	12 (42.9)	
When working with a patient with respiratory symptoms	Yes	246 (73.4)	135 (59.0)	20 (52.6)	< .001	342 (68.3)	43 (60.6)	15 (53.6)	< .001
	No	89 (26.6)	93 (41.0)	18 (47.4)		159 (31.7)	28 (39.4)	13 (46.4)	
When I am sick or showing respiratory symptoms	Yes	276 (82.4)	170 (74.9)	27 (71.1)	< .001	407 (81.2)	48 (67.6)	18 (64.3)	< .001
	No	59 (17.6)	57 (25.1)	11 (28.9)		94 (18.8)	23 (32.4)	10 (35.7)	
I never wear a mask	Yes	8 (2.4)	16 (7.0)	4 (10.5)	.02	17 (3.4)	7 (9.9)	4 (14.3)	.008
	No	327 (97.6)	211 (93.0)	34 (89.5)		484 (96.6)	64 (90.1)	24 (85.7)	

a Self-reported vaccination status.

b Derived from Pearson χ^2^.

c Included testing for all patients, regardless of symptoms.

During high-risk procedures such as collecting respiratory specimens, similar proportions reported masking whether they were up to date on COVID-19 vaccination (*n* = 136, 40.6%) or not (*n* = 85, 37.4%), and among those intending to receive the influenza vaccine (*n* = 196, 39.1%) compared to those who did not (*n* = 23, 32.4%). When entering rooms under droplet precautions, nearly three-quarters of respondents reported wearing masks, with rates ranging from 70.4% to 74.9%, regardless of COVID-19 or influenza vaccination status. When working with patients presenting with respiratory symptoms, 73.4% (*n* = 246) of COVID-19 vaccinated respondents reported masking compared to 59.0% (*n* = 135) of those unvaccinated, while 68.3% (*n* = 342) of those planning influenza vaccination masked compared to 60.6% (*n* = 43) who were not planning vaccination. Masking when personally ill or symptomatic ranged from 67.6% (*n* = 48) among those not intending to receive the influenza vaccine to 82.4% (*n* = 276) among respondents vaccinated against COVID-19. Very few respondents reported never wearing a mask (2.4% – 9.9%).

### Collection

An opened ended survey question solicited 211 additional comments. The top emergent themes were “masks are helpful” (*n* = 42, 19.9%), “against mask mandates” (*n* = 39, 18.4%), and “masks are not important or helpful” (*n* = 33, 15.6%).

## Discussion

Our study examines the postpandemic landscape of masking by exploring the beliefs, barriers, and perceptions surrounding mask usage among HCP involved in direct patient care. Overall, self-reported masking adherence rates remain low, highlighting a system wide gap in masking compliance. Although participants generally recognized that masking protects against respiratory infections, this belief did not consistently translate into practice. While 64.4% (*n* = 400) of participants recognized that masking protects others, suggesting confidence in its general effectiveness, this did not necessarily extend to a sense of social responsibility. Most disagreed with the statements that not wearing a mask endangered patients (*n* = 253, 40.5%), colleagues (*n* = 268, 42.9%), or family members (*n* = 269, 43%). This aligns with prior studies demonstrating a postpandemic decline in coronavirus risk perception and reduced protective behaviors.^17^ Support for masking policies also varied with the majority (*n* = 347, 53.0%) endorsing visitor masking in hospitals. Only 42.1% (*n* = 276) of participants endorsed routine masking during clinical encounters with registered nurses, AHPs, and those part of ancillary staff being least supportive. Lower masking support among nurses may reflect pandemic-related fatigue and burnout. As frontline caregivers for respiratory patients, nurses faced sustained strain, with 91.1% reporting prolonged burnout and 61.0% low job satisfaction.^20^ These factors likely contribute to resistance toward masking in non-mandated healthcare settings postpandemic and underscore a potential gap in understanding masking as a preventive infection control practice beyond symptomatic scenarios.

During the COVID-19 pandemic, HCP faced significant physical and psychological stressors.^18^ Most participants masked when entering patient rooms under droplet precautions but rarely during respiratory specimen collection, suggesting low perceived risk. Adherence was below 75% for all roles, except registered nurses, who may have higher compliance due to more patient contact, training or awareness of infection control protocols, and stronger accountability due to their frequent caregiving role. Other roles may perceive lower risk or face workflow constraints, reflecting a broader shift in HCP perceptions, with some clinical activities no longer seen as requiring consistent masking. In addition, because respiratory specimen collection often included testing asymptomatic patients as part of the hospital’s universal COVID-19 admission testing policy, some HCPs may have perceived themselves to be at lower risk of exposure.

Approximately half of participants in our study reported being up to date on COVID-19 vaccination. While prior studies have explored vaccine hesitancy—highlighting barriers such as safety concerns, mistrust of government, and perceived violations of autonomy—our focus was on the relationship between vaccination status and masking behaviors.^19^ While vaccination status (COVID-19 or influenza) and masking behavior differed between clinical scenarios, the absolute differences among vaccinated or not vaccinated were small. Consistent with prior studies examining infection prevention practices during the COVID-19 pandemic, masking behavior among HCPs did not differ meaningfully by vaccination status. This finding suggests that adherence to masking was primarily driven by institutional infection prevention policies and workplace norms rather than by individual vaccination decisions. Minor variations observed between groups may reflect contextual influences such as pandemic fatigue, perceived protection afforded by vaccination, and evolving public health messaging that, following vaccine rollout, shifted emphasis from universal precautions to individual responsibility. These factors may have contributed to subtle differences in masking behavior independent of vaccination status.^20–22^

During the pandemic, HCP often masked in part due to social norms.^23^ When asked who influenced their decision to mask, the most common response was “no one,” suggesting that masking decisions are now predominantly personal rather than guided by peers, friends, or family. This reflects a broader shift in postpandemic norms. Consistent with prior studies, our findings indicate that communication challenges, speech intelligibility issues, hearing impairment, and general discomfort remain common obstacles.^24–26^ Participants most frequently reported skin irritation, difficulty breathing, and vision interference, underscoring physical discomfort as a persistent deterrent. Open-ended responses revealed a wide spectrum of attitudes—ranging from “masks are helpful” to “masks are not important or helpful” and opposition to mask mandates—highlighting ongoing polarization around masking in clinical settings. A response highlighting opposition to mask mandates was “institutionwide mandatory masking is a net negative and interferes with important parts of the human experience.” These findings suggest that strategies to improve adherence must address both perceptual and practical barriers. Potential interventions include targeted education to reinforce the role of asymptomatic transmission, provision of masks with enhanced breathability and acoustic transparency, availability of communication aids, and reinforcement of risk-based masking guidance. Addressing these factors in tandem may help bridge the gap between knowledge, risk perception, barriers, and consistent protective behaviors in healthcare settings.

Our findings suggest declining trust in community-level transmission risk and reduced masking in clinical settings following the decline in COVID-19 cases. This behavior may be driven by a lower perceived threat of COVID-19 and pandemic fatigue. Education remains critical to reinforce that HARIs pose ongoing risks to both patients and HCP, emphasizing their continued prevalence and asymptomatic transmission. Masking should be promoted as one of several complementary strategies—alongside vaccination, ventilation, and hand hygiene—that together strengthen infection prevention and control efforts in healthcare environments.

Our study has several limitations. Vaccination status was self-reported and not validated against records, introducing potential misclassification. Participants may also have overreported vaccination due to social desirability, which could underestimate differences in masking behavior or inflate associations with vaccination. These factors should be considered when interpreting results. In addition, as a single-center study conducted at an academic medical center, our findings may have limited generalizability to other non-academic healthcare settings. The cross-sectional nature of the study precludes causal interference, and nonresponse bias may have affected sample representatives, as those with stronger views may have been more inclined to participate. Lastly, the timing of the survey—during peak respiratory virus season—may have affected participants’ attitudes and behaviors toward masking and vaccination.

In summary, we have shown that the overall perceived risk of acquiring a respiratory illness is low in the postpandemic era. There is a need to reinforce infection prevention as a shared responsibility, encompassing masking, vaccination, hand hygiene, and appropriate isolation practices. While masking remains an important component of this approach, educational efforts should also emphasize the broader set of measures that collectively reduce the transmission of HARIs.
